# The evolution of tumor metastasis during clonal expansion with alterations in metastasis driver genes

**DOI:** 10.1038/srep15886

**Published:** 2015-10-30

**Authors:** Kimiyo N. Yamamoto, Akira Nakamura, Hiroshi Haeno

**Affiliations:** 1 Department of Biology, Faculty of Sciences, Kyushu University, Fukuoka 812-8581, Japan; 2 Department of Radiation Oncology and Image-Applied Therapy, Kyoto University, Kyoto, Japan

## Abstract

Metastasis is a leading cause of cancer-related deaths. Carcinoma generally initiates at a specific organ as a primary tumor, but eventually metastasizes and forms tumor sites in other organs. In this report, we developed a mathematical model of cancer progression with alterations in metastasis-related genes. In cases in which tumor cells acquire metastatic ability through two steps of genetic alterations, we derive formulas for the probability, the expected number, and the distribution of the number of metastases. Moreover, we investigate practical pancreatic cancer disease progression in cases in which both one and two steps of genetic alterations are responsible for metastatic formation. Importantly, we derive a mathematical formula for the survival outcome validated using clinical data as well as direct simulations. Our model provides theoretical insights into how invisible metastases distribute upon diagnosis with respect to growth rates, (epi)genetic alteration rates, metastatic rate, and detection size. Prediction of survival outcome using the formula is of clinical importance in terms of determining therapeutic strategies.

Metastasis is the major cause of cancer-related deaths and an important clinical challenge^1^,^2^,^3^. Metastatic disease is commonly observed at the time of detection, although the frequency varies among cancer types; up to 60% in pancreatic and ovary cancers, but 20% in breast cancer^4^. Even in operable cases, surgical resection alone as a local control is sometimes inefficient due to recurrence in distant organs after treatment^2^. For example, a study reporting 25-year follow-up data determined that about 25% of breast cancers recurred at distant sites up to 20 years after treatment^5^, and ~15% of colon cancer patients who underwent open or laparoscopically assisted colectomy experienced recurrence after 3 years^6^. Because distant recurrence occurs even in non-metastatic disease upon diagnosis, it is possible that undetectable micro-metastases spreading to distant sites prior to detection will result in treatment failure. Thus, accurate predictions of metastatic properties upon diagnosis are of clinical importance.

Metastasis occurs by the completion of multistage processes^7^. Tumor cells invade the host stroma, penetrate blood vessels, and enter the circulation to produce new colonies in distant organs. The success of metastasis depends on intrinsic properties of the tumor and host environmental factors. Metastasis capability has recently been linked to specific genomic alterations in the primary tumor cells^8^. Deletion in a specific region of the genome promotes metastatic formation *in vivo* and *in vitro* among various cancers, indicating the existence of metastasis suppressor genes (MSGs)^9^. For example, the suppression of KLF17 expression promoted breast cancer cell invasion, and the *in vivo* suppression of a sialyltransferease (ST6GalNAc2) increased the number of metastatic lesions in mice^10^,^11^. Moreover, other metastasis-related genes—such as Slug, Snail, Goose-coid, Twist, and ZEB1—were identified that upregulation of these genes induced epithelial–mesenchymal transition (EMT). EMT alters the intrinsic properties of tumor cells, resulting in an increase in mobility and invasiveness^12^,^13^. Overall, deregulation of these genes drives the metastatic process.

Mathematical models have contributed to our understanding of metastasis. A logistic regression model was evaluated to predict the prognoses of testicular cancer patients utilizing several clinical characteristics, such as serum values of tumor markers and the total number of metastatic sites^14^. A model of competition between tumor and normal cells during periodically pulsed chemotherapy was designed to investigate the period of pulse and the amount of dose in which a tumor recurs due to the presence of a small number of metastatic cells^15^. In breast cancer, the correlation between a delay of surgery and the risk of distant metastases was investigated^16^; a delay in surgery increased the risk of metastasis by 1–4% per month. Iwata *et al.* added the colonization effect and the fractal dimension of blood vessels infiltrating the tumor to the model and fitted it to the clinical data from liver cancer patients^17^. The dynamic interactions between a primary tumor and metastases were investigated when early metastasis from the primary tumor occurred^18^. The expected number of metastatic cells was investigated when a single or two mutations were necessary to confer metastatic abilities to a cell^19^,^20^. A branching process model of tumor metastases was developed to investigate the relationship between the growth rate of the primary tumor and the export^21^. More recently, Haeno and Michor considered a single alteration conferring the metastatic ability to a primary tumor cell during clonal expansion and calculated the expected number of metastatic sites and cells and survival time in autopsy studies^22^. The model describes cancer progression where activation of EMT-promoting genes is responsible for metastatic propensity. However, there is another mechanism in which inactivation of MSGs confers metastasis ability to cells. In this case, alterations in two alleles are required for export of primary cells. Moreover, in practical cases, it is possible that both activation of EMT-promoting genes and inactivation of MSGs are involved in metastasis formation, which should be considered in mathematical models of the evolution of metastasis.

In this report, we design and analyze a stochastic mathematical model of the evolution of tumor metastases caused by genetic alterations in an expanding cancer cell population. First, we derive formulas for the probability of metastasis and the distribution of the number of metastatic sites at a specific time under the condition that two genetic alterations confer metastatic ability to primary cells. We then investigate practical disease progression in cases in which both EMT-related genes and MSGs are responsible for metastasis formation. Finally, we derive the mathematical formula for survival time based on the model. The formula is validated by comparing it to clinical data of pancreatic cancer patients with/without metastasis, as well as computer simulations. The model provides theoretical insights into how invisible metastases distribute at the time of detection with respect to growth rate, (epi)genetic alteration rates, metastatic rate, and detection size. Successful prediction of survival outcomes will support treatment decision-making in clinical settings.

## Results

### The model

**Figure 1:**
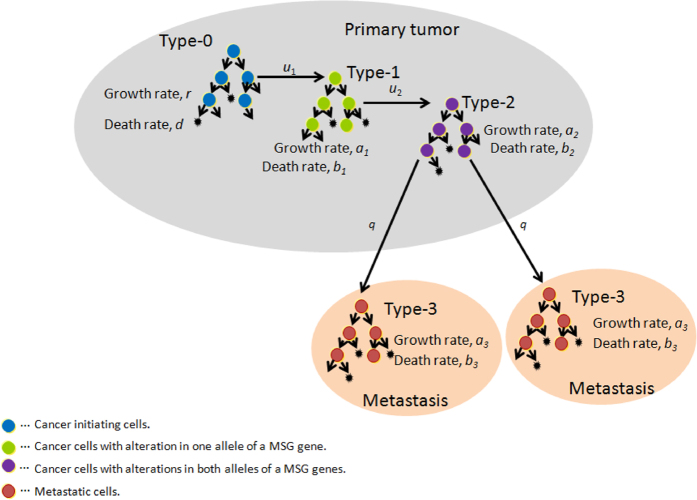
Schematic illustration of the model in which inactivation of MSGs is required for the acquisition of metastatic ability. Cancer-initiating cells that have not yet evolved the ability to metastasize are termed type-0 cells. Type-0 cells give rise to type-1 cells through an alteration in one allele of an MSG with probability *u*_1_ per cell division. Type-1 cells give rise to type-2 cells through another alteration in the other allele of the MSG with probability *u*_2_ per type-1 cell division. Type-2 cells have metastatic ability due to loss of function of an MSG. Once a type-2 cell has been produced, it has a certain probability of emigrating to distant organs, which is denoted by *q*. The metastasized cells at distant sites are referred to as type-3 cells. The total number of tumor cells upon diagnosis is denoted by *M*. Cancer cells divide at rate *r*, *a*_*1*_, *a*_*2*_, and *a*_*3*_ and die at rate *d*, and *b*_*1*_, *b*_*2*_, and *b*_*3*_, per unit time for type-0, -1, -2, and -3 cells, respectively.

Consider an exponential growth of cancer cells starting from a single cell in the primary site. The initiating population does not have the potential for dissemination, but eventually obtains it by inactivating two alleles of a metastasis suppressor gene (MSG). Cells with metastatic ability can establish metastatic sites; these started from a single metastatic cell. Cells at primary and metastatic sites follow a stochastic process: in a unit time, cells may divide (with a possibility of alteration), die, metastasize, or do nothing. When the total number becomes sufficiently large, the disease is diagnosed (Fig. 1).

Initiating tumor cells that have not yet evolved the ability to metastasize are called type-0 cells. These cells divide at rate *r* and die at rate *d* per unit time. Type-0 cells give rise to type-1 cells through an alteration in one allele of an MSG with probability *u*_1_ per type-0 cell division. Type-1 cells divide and die at rates of *a*_1_ and *b*_1_ per unit time. Since type-1 cells have one more intact allele of the MSG, type-1 cells are unable to metastasize. Type-1 cells give rise to type-2 cells through another alteration in the other allele of the MSG with probability *u*_2_ per type-1 cell division. Type-2 cells have metastatic ability by a consequence of loss of function of an MSG. These cells divide at rate *a*_2_ and die at rate *b*_2_ per unit time. Once a type-2 cell has been produced, it has a certain probability of being exported from the primary tumor to attempt to establish metastases at another location. The integrated rate of leaving the primary site and founding a new colony at a distant site is denoted by *q*. Once a type-2 cell has emigrated to a distant site, it may go extinct or grow exponentially with a division and death rate of *a*_3_ and *b*_3_ per unit time, respectively. Metastatic cells at distant sites are referred to as type-3 cells. The total number of tumor cells (including all four types) upon diagnosis is denoted by *M* (Fig. 1).

### Probability and expected number of metastatic sites

Let us first consider the expected number of mutations to produce type-1 cells from *w* type-0 cells, *R*_*w*_. Although the quantity has already been previously derived^23^, we will briefly show the derivation. Let 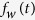 be the probability that type-0 cells is *w* at time *t*. Then we have


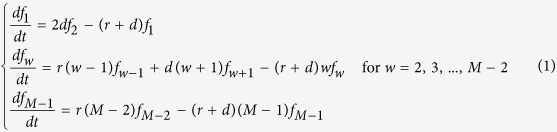


The initial condition is 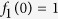, and 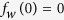 for *w* = 2, 3, …, *M *− 1. Before the number of type-0 cells reaches 0 or the detection size *M*, there are chances to produce type-1 cells by mutations. The expected number of mutational events when there are *w* type-0 cells is obtained from the product of the mutation rate of *w* type-0 cells and the total time of being *w* type-0 cells. Then we have


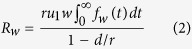


Here, 1-*d*/*r* represents the probability that the type-0 cells do not go extinct. We consider the number of mutational events conditional to the survival of the type-0 population. With 
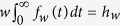
, Eq. (1) becomes


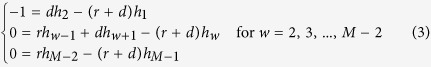


By solving Eq. (3) and assuming *M* is sufficiently large, we have 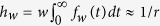. Please see Iwasa *et al.* (2006) for the detail of the derivation^23^. Then, the expected number of mutational events when there are *w* type-0 cells is given by


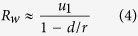


Finally, the probability that the first mutation occurs when there are *w* type-0 cells, *P*_*w*_, is given by


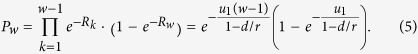


Here, the first exponent, 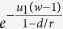, represents the probability that no successful type-1 lineage appears until the total size becomes *w*, assuming the number of mutational events of *w* type-0 cells follows a Poisson distribution with mean *R*_*w*_. The second factor represents the probability that a successful type-1 lineage emerges when there are *w* type-0 cells. See Iwasa *et al.* (2006) and Haeno *et al.* (2007) for the detailed derivation of equation (5)^23^,^24^. The maximum abundance of type-1 cells produced by *w* type-0 cells upon diagnosis, 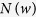, is given by 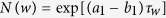, assuming exponential growth of type-1 cells without additional mutations. Here 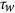 is calculated by the following equation:


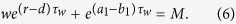


Once a type-1 lineage emerges, the expected number of mutations that produce a type-2 lineage, *R*′_*x*_, is given by


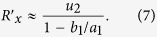


Eq. (7) is obtained from the same method of derivation as Eq. (4). Then the probability that the first mutation occurs when there are *x* type-1 cells, a lineage of which is produced from *w* type-0 cells, *P*_*x*_ is given by


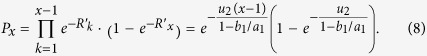


Finally, the probability that metastatic cells exist at a certain detection size, *P*, is given by


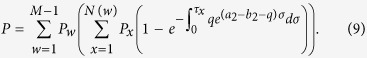


Here, the last factor, 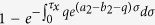, represents the probability of the metastatic event from type-2 cells during 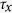. The time period from the emergence of a type-2 cell to diagnosis, 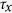, is calculated from the following equation:


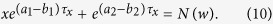


Let us next investigate the expected number of metastatic sites with more than *z* cells from a type-2 lineage at the detection time, 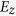. Assuming exponential growth of type-3 cells after the emergence, the time period until reaching *z* type-3 cells from one type-3 cell, 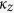, is given by 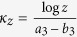. Then, the number of metastatic sites with more than *z* cells is considered as the number of metastatic events at the time of which there is sufficient time, 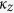, to increase up to *z* type-3 cells until diagnosis. Then, the expected number of metastatic events that will produce metastases with more than *z* type-3 cells when the total number of type-1, -2, and -3 cells becomes *N*(*w*) conditional on starting with *x* type-1 and one type-2 cells, *S*, is given by


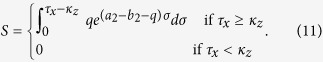


Note that 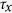 has already been calculated in Eq. (10). Finally, the expected number of metastatic sites with more than *z* cells from a type-2 lineage is given by


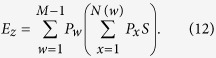


The expected number of metastatic sites the cell number of which is between 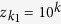 and 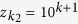 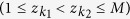, and is given by


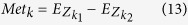


### Expected number of each cell type upon diagnosis

Let us next derive the expected number of cells in type-1 (*E*_1_), -2 (*E*_2_), and -3 (*E*_*3*_) populations at the time of diagnosis. Please see Eq. (S13) and Eq. (S17) in Yamamoto *et al.* (2014) for the detailed expression of *E*_1_ and *E*_*2*_^25^. To obtain *E*_3_, 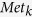 in Eq. (13) is rounded off into 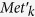, we draw random numbers 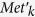 times from a uniform distribution between 10^*k*^ and 10^*k*+*1*^ and sum the numbers from the case of *k* = 0 to *k* = *K* (<log_10_*M*). Furthermore, we analyzed the results of the distributions of the number of metastatic sites in different size categories using direct computer simulations (System 21–26). We then heuristically found that they were exponentially distributed with the mean of the expected number in each category (Fig. S1).

### Metastatic properties in cases in which cancer cells acquire metastatic ability through two alterations

**Figure 2:**
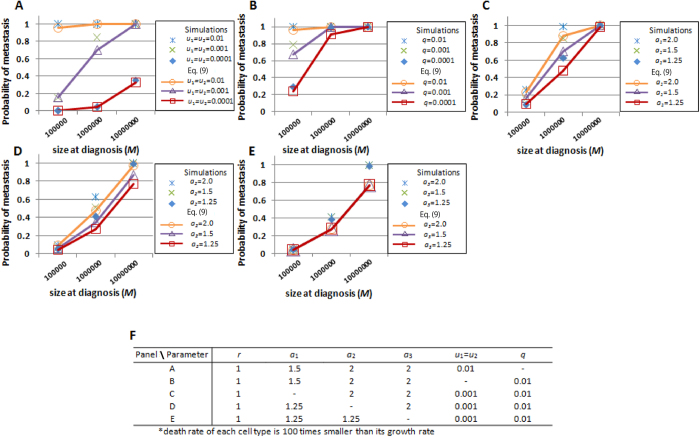
Probability of metastasis generated from type-2 cells. The figures show the dependence of the probability of the existence of metastatic cells (type-3 cells) upon diagnosis for various parameters. The dots show the results of the direct computer simulations, while the lines show the predictions of the analytical approximations (Eq. (9)). Parameter values used are listed in panel F.

We initially tested the accuracy of the formulas for the existence probabilities of metastatic sites with a wide space of parameter values of genetic alteration rates (*u*_1_ and *u*_2_), dissemination rates (*q*), the detection size of tumor (*M*), and growth rates of primary and metastatic cancer cells (*a*_1_, *a*_2_, and *a*_3_) (Figs 2, S2 and S3). The analytical approximation Eq. (9) accurately predicted the results of the exact stochastic computational simulations. The increase in (epi)genetic alteration rates, metastatic rate, the detection size, and growth rates of type-1 and type-2 cells at the primary site led to the increase in the existence probabilities in any parameter set (Fig. 2A–D). By contrast, the increase in growth rates of metastases *a*_3_ did not affect the probabilities (Fig. 2E).

**Figure 3:**
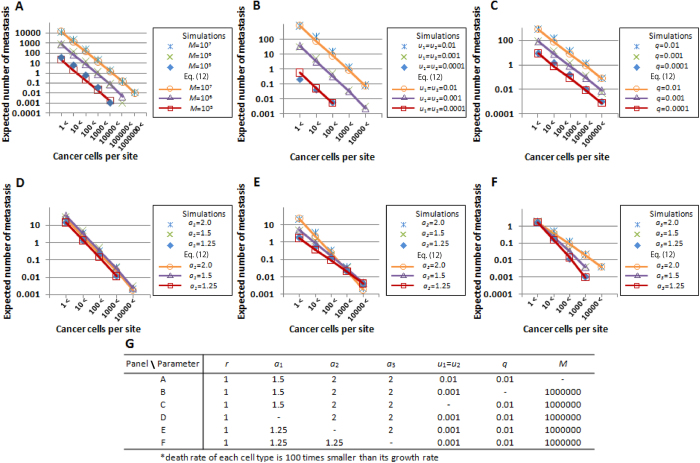
Expected number of metastases generated from type-2 cells. The figures show the dependence of the expected number of metastatic sites upon diagnosis on various parameters. The dots show the results of the direct computer simulations, while the lines show the predictions of the analytical approximations (Eq. (12)). Parameter values used are listed in the panel G.

Next, we tested the accuracy of the formula for the expected number of metastatic sites where the size of each metastasis is over z 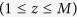 (Figs 3, S4 and S5). The analytical approximation Eq. (12) accurately predicted the results of the simulations with a wide space of *u*_1_, *u*_2_*, q*, and *M*, and with various growth rate sets in cancer cell types (Fig. 3). As observed in the investigation of the probabilities of metastatic existence, the increase in alteration rates *u*_1_ and *u*_2_, metastatic rate *q*, and detection size *M* resulted in an increase in the expected number of metastatic sites (Fig. 3A–C). Interestingly, the expected number decreased when the growth rate of type-1 cells was sufficiently high (Fig. 3D). Growth rates of type-2 cells *a*_2_ and metastases *a*_3_ increased the expected number of metastatic sites (Fig. 3E,F). Here, we found that the number of small metastases was significantly larger than that of large metastases in any parameter set. For example, there were almost 10-fold differences in the expected numbers when the number of cancer cells per site increased by 10-fold (Fig. 3).

### Metastatic properties in cases in which cancer cells acquire metastatic ability through one and two (epi)genetic alterations

**Figure 4:**
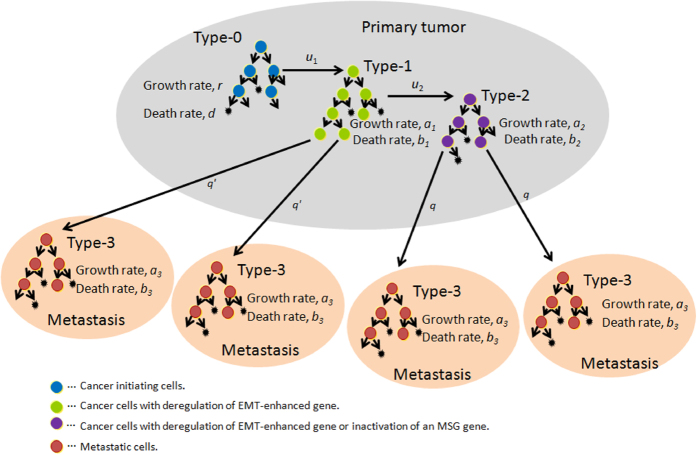
Schematic illustration of the model in which cancer cells obtain metastatic propensity by alterations in MSGs and EMT-related genes. In addition to type-2 cells, type-1 cells have the ability to generate metastatic cells (type-3 cells). The emigration rate from type-1 cells to generate type-3 cells is denoted by 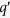.

In this section, we investigated metastatic progression following tumor cells obtaining metastatic propensity by alterations in MSGs and EMT-related genes. The schematic of the mathematical framework is shown in Fig. 4. In previous studies under the condition that one genetic alteration is responsible for metastasis^22^, we derived equations for the probability of metastasis and the expected number of metastatic sites (Eq. (2) and Eq. (3) in Haeno and Michor (2010)). By extending the above theory, we can investigate the probability, the expected number, and the distribution of metastases when one and two (epi)genetic alterations are responsible for metastatic events.

In this formulation, metastatic events from type-1 cells are allowed at rate 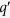 . Let us first investigate the expected number of metastatic sites with more than z cells from a type-1 lineage at the detection time, 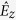. It is given by


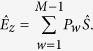


Note that *P*_*w*_ is given in Eq. (5). Here, the expected number of metastatic events that will produce metastases with more than z type-3 cells when the total number of type-0, -1, and -3 cells becomes *M* conditional on starting with *w* type-0 and one type-1 cells, 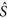, is given by


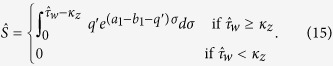


Note that the time period from one to z type-3 cells, 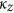, is shown in the previous section. The time period from the emergence of the type-1 cell to diagnosis is denoted by 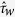, and is calculated from the following equation:


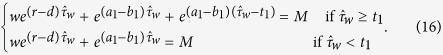


The first metastatic cell (type-3 cell) is expected to appear at time *t*_1_ after the emergence of a type-1 lineage, which is calculated from the following equation: 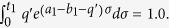


The expected number of metastases generated from type-1 cells, the abundance of which is between 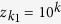 and 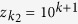 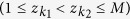, is given by


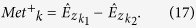


Finally, with Eq. (13), the expected number of metastatic sites generated from cancer cells with both one and two genetic alterations, the abundance of which is between 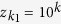 and 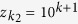 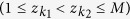, is given by


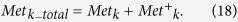


To obtain the total expected number of type-3 population at diagnosis, *E*_3_total_, 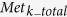 in Eq. (18) is rounded off into 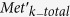, we draw random numbers 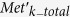 times from a uniform distribution between 10^*k*^ and 10^*k*+*1*^ and sum the numbers from the case of *k* = 0 to *k* = *K* (<log_10_*M*).

**Figure 5:**
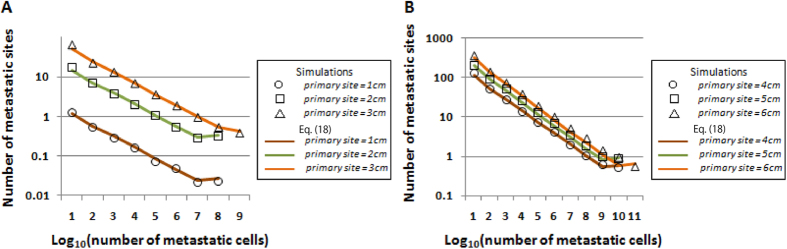
Expected number of metastases generated from both type-1 and type-2 cells in different size categories. The number of metastatic sites of the indicated sizes upon diagnosis on the *x*-axis by computational simulation (dots) and theoretical formulas (lines) when the primary tumor is diagnosed at (**A**) 1–3 cm and (**B**) 4–6 cm in diameter. Parameter values used are *r* = 0.11, *d* = 0.01*r, a*_*1*_ = 0.16, *b*_*1*_ = 0.01*a*_*1*_*, a*_*2*_ = 0.24, *b*_*2*_ = 0.01*a*_*2*_*, a*_*3*_ =* *0.58, *b*_*3*_ = 0.01*a*_*3*_*, u*_1_* *=* u*_2_ = 6.31×10^−5^, and *q *=* q*′ = 6.31×10^−7^, which are based on the estimation using time series clinical data in the pancreatic cancer autopsy program.

We examined the accuracy of the formulas using practical parameter values obtained from the pancreatic autopsy program^26^. We found that an MSG, such as TP53, and an EMT-related gene, such as SMAD4, are associated with metastatic propensities in pancreatic cancer^27^. We confirmed a good fit between Eq. (18) and the results of the modified computational simulations when metastatic events from type-1 cells are allowed at rate 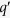 (Figs 5 and S6). We again observed an increase in detection size resulting in the increase in the expected numbers of metastatic sites (Figs 5 and S6). We also found that even in cases in which the total number of tumor cells upon diagnosis was about 10^8^, which represents an approximately 1-cm diameter primary tumor using a conversion equation from the number of cells to tumor volume (10^9^ cells occupy a volume of 1 cm^3^) with the assumption of spherical shape and 80% existence of stromal cells, the expected number of metastasis was over one (Fig. 5A). The number of metastases generated from type-1 cells is greater than generated from type-2 cells (Table 1 and Fig. S7). Markedly, there are more than 10-fold differences between the two when the number of cancer cells per site is over 10^7^, which is a clinically detectable size (Table 1 and Fig. S7).

**Figure 6:**
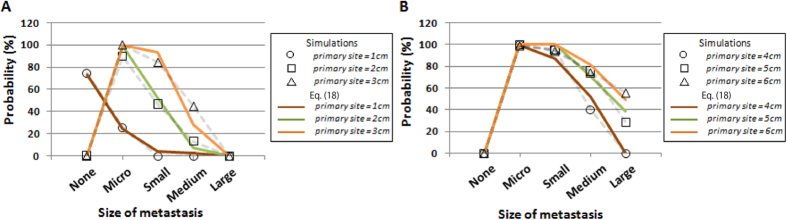
Probability of metastasis generated from both type-1 and type-2 cells in different size categories. Panels show the probabilities of existence of metastases of the indicated sizes upon diagnosis on the *x*-axis, with the assumption that the number of metastatic sites follows an exponential distribution with the mean of the expected numbers of the categories. “Large” denotes a metastatic site larger than 2.5 cm, “medium” denotes between 0.5 cm and 2.5 cm, “small” denotes between 0.1 cm and 0.5 cm, and “micro” denotes less than 0.1 cm. The diameter of the primary tumor upon diagnosis is (**A**) 1–3 cm and (**B**) 4–6 cm. Parameter values used are based on the estimation in the autopsy program: *r* = 0.11, *d* = 0.01*r, a*_*1*_ = 0.16, *b*_*1*_ = 0.01*a*_*1*_*, a*_*2*_ = 0.24, *b*_*2*_ = 0.01*a*_*2*_*, a*_*3*_* *=* *0.58, *b*_*3*_ = 0.01*a*_*3*_*, u*_1_* *=* u*_2_ = 6.31×10^−5^, and *q* = *q*′ = 6.31×10^−7^.

We then examined the accuracy of the existence probabilities of metastatic sites with various clinically plausible sizes upon detection. Let us first investigate the distributions of the number of metastatic sites of different size categories. We heuristically confirmed that they followed an exponential distribution with the mean of the expected numbers of categories shown in Eq. (18) (Fig. S8). Note that we regrouped metastatic sites into “None”, “Micro”, “Small”, “Medium”, and “Large” metastases according to their size. We observed a good fit between the results of estimations and the computational simulations (Figs 6 and S9). The increase in size of primary tumor upon detection resulted in an increase in the existence probabilities of metastases of greater size, which indicated that cases with metastases of greater size are more likely to be diagnosed as metastatic disease (Fig. 6). Markedly, the results suggested that micro-metastases exist in almost 100% of cases in which the primary tumor diameter at diagnosis was more than 2 cm (Fig. 6). In cases in which a primary tumor was 1 cm in diameter, approximately 20% of patients held metastases (Fig. 6A).

### Prediction of survival duration of cancer patients

Let us finally consider the tumor dynamics after diagnosis with treatment. There are two treatment options in accordance with the cancer guidelines National Comprehensive Cancer Network (NCCN) in the mathematical framework: (i) one may receive surgery followed by chemotherapy or (ii) one may receive only chemotherapy. A patient may not develop detectable metastatic sites upon diagnosis. In this case, we perform surgical resection and remove *ε* primary tumor cells at the time of diagnosis. We also consider the postoperative immunosuppression effect *α*, which increases the growth rate of each cell population for one month. The reduction in growth rate due to chemotherapy is denoted by *γ*. Note that equations for the expected number of cells in type-1 -2, and -3 populations at the time of diagnosis have already been given by *E*_*1*_, *E*_*2*_, and *E*_3_*total*_. The number of cells in type-0, -1, -2, and -3 populations at the end of the acute phase after surgery are denoted by *A*_0_, *A*_1_, *A*_2_, and *A*_3_, and these quantities are given by: 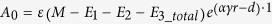, 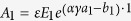, 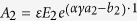, and 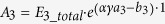. for type-0, −1, −2, and −3 populations, respectively.

Finally, the survival time, *t*_*s*_, when surgical resection is performed is calculated from the following equation:


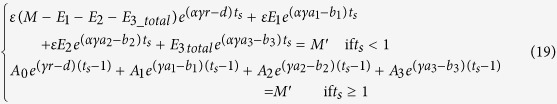


Here, we assume that once the total number of tumor cells exceeds 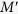, a patient dies. If a patient has metastatic sites upon diagnosis, only chemotherapy is given as a therapeutic intervention, and the survival time, *t*_*s*_ is given by


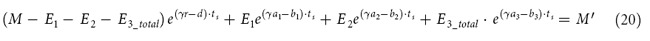


**Figure 7:**
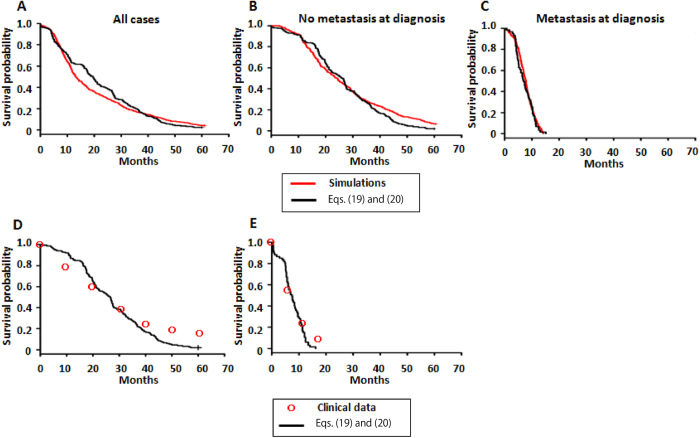
Overall survival based on computational simulations, theoretical predictions, and clinical data. Survival analyses comparing theoretical predictions (black lines, Eqs. (19) and (20)) and computational simulations (red lines) in cases of (**A**) the entire cohort, (**B**) those without, and (**C**) with diagnostic metastases. The numbers of simulation trials in simulations and equations are (**A**) 211 and 413, (**B**) 256 and 153, (**C**) 160 and 67, respectively. *P* values were (A) 0.506, (B) 0.201, and (c) 0.544, respectively. Panels D and E show a comparison of survival between the theoretical predictions and clinical data from the literature^28^,^29^ ((**D**) Oshima *et al.*, 2013, (**E**) Cunningham *et al.*, 2009). Parameter values used in theoretical predictions and computational simulation are based on the estimations in the autopsy program; *r* = 0.11, *d* = 0.01*r, a*_*1*_ = 0.16, *b*_*1*_ = 0.01*a*_*1*_*, a*_*2*_ = 0.24, *b*_*2*_ = 0.01*a*_*2*_*, a*_*3*_ = 0.58, *b*_*3*_ = 0.01*a*_*3*_*, u*_1_ = *u*_2_ = 6.31×10^−5^, *q* = *q′* = 6.31×10^−7^, *M* = 10^*N*(9.47,0.59)^, and 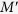 = 10^11.2^. Here, *N*(9.47, 0.59) represents the normal distribution with mean 9.47 and variance 0.59.

We investigated survival times with a specific parameter set obtained from the pancreatic cancer cohort. We confirmed good agreement between the predictions based on theoretical formulas and computational simulations in (i) all cases, (ii) cases without metastasis at diagnosis, and (iii) cases with metastasis (Fig. 7A–C). The results reproduced the clinical data from the literature within the estimated parameter values using clinical modalities (Fig. 7D,E)^26^. Importantly, we reproduced survival outcomes in not only patients with any clinical stages but also subpopulations of patients with operable cancer upon diagnosis and subpopulations with metastases upon diagnosis (Fig. 7D,E).

Parameter values used for the analysis were estimated in the previous work^26^. The net growth rates of primary and metastasis was estimated by using time series data of tumor sizes measured by computed tomography from 128 pancreatic patients^26^. The mean net growth rates of primary and metastatic tumor were 0.16 and 0.58 per month, respectively. The mean numbers of tumor cells at diagnosis (*M*) and at death (*M*′) were *M* = 10^9.47^, and *M*′ = 10^11.2^ by their imaging data, respectively. Note that the number of cancer cells in 1cm^3^ tumor bulk was assumed to be a billion. The rate of (epi)genetic alteration event that enables cells to metastasize and the metastatic rate were estimated by using information regarding the numbers of metastatic sites and metastatic cells at autopsy from the patient cohort. More precisely, the best combination of mutation and metastatic rates that achieve the best fit between the data of metastatic sites and metastatic cells and the predictions of their mathematical model were selected. The mutation and metastatic rates were 6.31 × 10^−5^ per cell division, 6.31 × 10^−7^ per month, respectively.

The results strongly support the validity of our model in terms of metastatic progression. The median OS (mOS) of operable cases was 25.51 months; on the other hand, the mOS of cases with metastasis was 7.43 months, which indicated that the survival outcome of cases with diagnostic metastases was significantly worse than that of cases without metastases (Fig. 7B,C). Furthermore, the most important prognostic factor was the total number of metastatic cells (Fig. S10). In addition, we validated the accuracy of the prediction using various parameter values (Fig. S11).

## Discussion

Although advances have been made in terms of reducing mortality rates, most deaths from cancer are due to metastatic disease. Considering the accumulating evidence on the genetic alterations related to metastasis, the evolutionary dynamics of metastatic progression with genetic alterations in metastasis suppressor genes (MSGs) and EMT-promoting genes should be interpreted in a mathematical model. In this study, we investigated the evolutionary dynamics of metastasis under the condition that two genetic alterations are responsible for metastasis. We have derived formulas for (i) the probability of metastasis, (ii) the expected number of metastatic sites, and (iii) the distribution of the number of metastatic sites. Moreover, we heuristically found that the number of metastases was exponentially distributed. We then investigated the pancreatic cancer progression that inactivating MSGs and alterations in EMT-promoting genes confer metastatic ability to primary cells using parameter values obtained from the pancreatic autopsy program^26^. Finally, we predicted the survival time of patients by employing the formulas. The framework was validated using clinical data^28^,^29^ as well as multiple runs of direct computational simulations based on the established model.

A clinically important question is how we obtain information on the entities of undetectable micro-metastases spreading to distant sites prior to their detection, which sometimes causes treatment failure. To answer this question, we obtained accurate predictions of metastatic properties as tested by computer simulations under the condition that two alterations are responsible for metastatic propensity. The probability and the expected number of metastasis are large when: (i) alteration rates are high; (ii) metastatic rates are high; and (iii) the size at which primary sites are detected is large (Figs 2, 3, and S2–S5). Here, we observed that a change in growth rates in metastatic organs *a*_3_ did not affect the probabilities (Fig. 2E). This is because each stochastic event of metastases is dependent on the number of metastatic-capable cells, which are cells in primary sites but not in metastatic organs. We also observed an excessive increase in the growth rate of type-1 cells *a*_1_ reduced the expected number of metastases (Fig. 3D). This occurs because the enhanced growth rate of type-1 cells decreases the time to reach a detectable size and reduces the probability of metastasis.

Another clinically significant result in this study is prediction of the survival outcome of cancer patients undergoing various therapies. Here, we derived analytical approximations that reproduced both clinical data and direct computational simulations (Fig. 7). The survival outcome of cases with diagnostic metastases was significantly worse than that of cases without metastases (Fig. 7B,C), which was explained by the result that the number of metastatic cells is the most significant prognostic factor (Fig. S10). Therefore, progress in the management strategies of metastatic patients, such as a combination of chemotherapies, is urgently required to improve patient survival.

By adopting clinically relevant parameter values, patients can be anticipated to have a substantial number of metastatic cells upon diagnosis (Fig. 5). Even in cases with a primary tumor of 1-cm diameter, some patients held metastases (Fig. 6A), and the expected number was greater than one (Fig. 5A). Micro metastases, most of which are likely undetectable using clinical imaging devices, exist in almost 100% of cases in which the primary tumor diameter upon diagnosis was more than 2 cm, indicating that the detectable metastases are just the “tip of the iceberg” (Fig. 6). Unfortunately, the number of metastatic cells (but not primary tumor cells) was significantly associated with survival duration (Fig. S10). Therefore, regardless of the attainability of complete primary site resection, clinicians should consider spreading lesions in distant organs when making patient management decisions. At the same time, the results suggested a correlation between tumor size and the likelihood of metastases (Fig. 6), and almost 80% of cases with a primary tumor 1-cm diameter have no metastasis and the possibility of achieving complete eradication of cancer cells by surgical resection (Fig. 6A). These results indicate the necessity of early detection in any type of cancer.

One interesting observation is that the number of metastatic sites generated from type-1 cells is greater than generated from type-2 cells (Table 1 and Fig. S7). Markedly, there were greater than 10-fold differences between the two when the number of cancer cells per site was >10^7^, a clinically detectable size (Table 1 and Fig. S7). These results suggest the importance of distinguishing the responsible alterations in MSGs and EMT-promoting genes in designing a mathematical model of metastasis progression (Figs 1 and 4). In fact, the proportion of cases in the metastatic stage upon diagnosis varies among cancer types. In some cancer types—such as breast cancer, prostate cancer, or melanoma of the skin—patients are likely to have localized or regional cancer, but not distant cancer, at the time of diagnosis^4^. Such a stage distribution among cancer types may be explained in part by the number of (epi)genetic alterations required for acquisition of metastatic ability. In melanoma, overexpression of ZEB1 and TWIST1 with low expression of ZEB2 significantly shortens metastasis-free survival^30^. The consideration of metastatic propensity only in type-2 cells would be appropriate in these types of cancer. By contrast, in colorectal cancer, in which a relatively large proportion of patients are diagnosed at a distant stage, an APC alteration alone activates EMT, leading to high invasive behavior^31^. A model considering metastatic propensity in both type-1 and type-2 cells would be appropriate for these types.

Our mathematical framework incorporating mutational profiles of responsible genes of metastasis could partly unveil one important hallmark of cancer – metastasis. Recent genomic studies have revealed specific mutational patterns responsible for the cancer hallmarks such as angiogenesis, abnormal metabolic process, capability to evade the immune system, the extent of genome instability and inflammation^32^. In future, by incorporating the mutational profiles of each cancer hallmark considering interactions between hallmarks, our mathematical model will be applicable to investigate the complex process of cancer evolution.

In this study, we assumed a simple exponential growth mode without intercellular competition based on density effects. Based on our investigation of metastatic progression in practical cases in which both MSGs and EMT-promoting genes confer metastatic ability to primary cells, we used parameters obtained from the pancreatic cancer autopsy program and showed the probability, the expected numbers, and the distribution of the number of metastatic sites^26^. The model explained pancreatic carcinogenesis and patient survival (Fig. 7D,E). In future, the model will be able to predict the metastatic profiles of other type of cancers, provided that growth and death rates, export rate from the primary site, and (epi)genetic alteration rates, as well as molecular discoveries regarding alterations leading to enhanced metastatic propensity, are identified. In addition, we selected treatment effects following the current cancer guidelines. By introducing treatment strategies which have not reached clinical consensus into the mathematical model, we can evaluate the effects of therapies. In summary, our approach for the evaluation of invisible metastatic distribution upon detection provides novel insight into disease progression upon diagnosis, and the accurate prediction of survival outcome of patients using analytical formulas support the subsequent determination of treatment strategies.

## Methods

### Computer simulations

We performed exact computer simulations of the stochastic process. There are four types of cell: type-0, type-1, type-2, and type-3 cells. Their respective numbers are denoted by *w*, *x*, *y*, and *z*_*i*_; the latter specifies the number of cells in the *i*-th metastatic site. A change in *w*, *x*, *y*, and *z*_*i*_ can occur by cell division (with a possibility of alteration), cell death, or export from the primary site. An export event means the establishment of a new metastatic colony starting from one immigrant cell. The total number of sites where tumor cells can found metastases is denoted by *I*. Initially, there is one type-0 cell, *w* = 1, and no type-1, -2, or -3 cells for all *i* *Ε* *I*.

Let us consider that all possible events in the stochastic simulation consist of the production and death of a type-0, -1, -2 or -3 cell, and the export of a type-2 cell to a new metastatic site. Under the condition that one event occurs, the probability of each event is given by its rate normalized to the sum of the rates of all possible events, given by 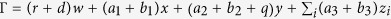. The timing of one event is given by an exponential distribution with mean 1/Γ. Each event and the timing of the event is stochastically chosen by pseudo random numbers generated by Mersenne Twister algorithm^33^. This procedure of the stochastic simulations is based on Gillespie’s algorithm^34^. The process is continued either until all cells go extinct, 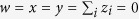 or until the total cell number reaches the final size, 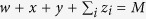, upon diagnosis.

The transition probabilities between states in the stochastic simulation are determined as follows. The number of type-0 cells increases if a type-0 cell divides without mutating. Hence, the probability that the number of type-0 cells increases by one is given by


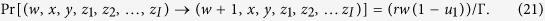


The number of type-1 cells increases by one if alteration of a type-0 cell or division of a type-1 cell without mutation occurs. It is given by


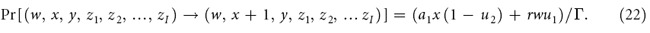


The number of type-2 cells increases by one if alteration of a type-1 cell or division of a type-2 cell occurs. It is given by


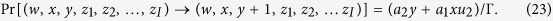


Export of type-2 cell to a new metastatic site (*j *
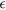
* I*) increases the number of metastatic cells at site *j* by one and decreases the number of type-2 cells by one. It is given by


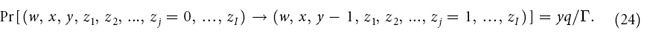


The probability that the number of type-3 cells in the *i*-th metastasis increases by one by division is given by


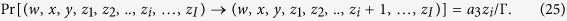


The probabilities that the numbers of type-0, -1, -2, and *i*-th type-3 cells decrease by one are given by


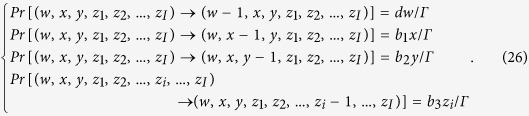


For each parameter set, we perform many independent runs of the stochastic process to account for random fluctuations. We count the fraction of runs that reach the final size, *M*. We also record the number of metastatic sites with non-zero cell numbers, and the number of type-3 cells in each metastatic site, from which the dispersal pattern of metastasis upon diagnosis can be inferred.

To investigate survival duration in each simulation case, the process is continued until the total number of cells reach *M*′, which is defined as the total number of tumor cells at the time of patient’s death. Each case of the stochastic simulation receives either of two treatment options according to NCCN guideline. If no metastases are detected, the case is regarded as non-metastatic disease. In this case, surgical resection is performed followed by chemotherapy. If metastases are detected, only chemotherapy is administrated. We define the diagnostic accuracy for the assessment of the existence of metastases by medical devices as follows. The detection probability of metastases increases as those sizes increase. Each metastasis is supposed to be detected with a probability of zero when its diameter is less than 1 cm, with a probability 0.8 if its diameter is between 1 cm and 3 cm, and with a probability 1.0 if its diameter is more than 3 cm. Surgical resection removes *ε* fraction primary tumor cells at diagnosis. In this case, the postoperative immunosuppression effect, *α*, is followed, which increased the growth rate of any cell population for one month. Chemotherapy reduces growth rate of any cell type by *γ*. The survival duration of one simulation run was measured by the sum of the time of all stochastic events the run experienced from diagnosis, at which the total number is *M,* to the death, at which the total number is *M*′

### Statistical analysis

The overall survival was defined as the time from the primary surgery to death attributed to any cause. The survival rates were estimated using the Kaplan-Meier method. Survival data were analyzed using the log-rank test. All *P* values less than 0.05 were considered to indicate statistical significance. All statistical analyses were performed using R version 3.1.0, by the R foundation for statistical computation.

## Additional Information

**How to cite this article**: Yamamoto, K. N. *et al.* The evolution of tumor metastasis during clonal expansion with alterations in metastasis driver genes. *Sci. Rep.* **5**, 15886; doi: 10.1038/srep15886 (2015).

## Supplementary Material

Supplementary Information

## Figures and Tables

**Table 1:** The number of metastatic sites generated from type-1 and type-2 cells (size upon diagnosis: 3 cm).

Number of cells per site	Number of metastases from type-1	Number of metastases from type-2
1–10	44.10934641	6.053571
10^1^–10^2^	21.52344329	2.141703
10^2^–10^3^	11.9642442	0.873389
10^3^–10^4^	6.370681267	0.338793
10^4^–10^5^	3.377077836	0.130736
10^5^–10^6^	1.789373054	0.050423
10^6^–10^7^	0.948071671	0.019446
10^7^–10^8^	0.502318869	0.0075
10^8^–10^9^	0.266144576	0.002892
10^9^–	0.407156463	0.004008
