# Supplementary material for: The evolution of tumor metastasis during clonal expansion with alterations in metastasis driver genes
Source: Sci Rep. 2015 Oct 30;5:15886. doi: 10.1038/srep15886 (PMC4626809; doi:10.1038/srep15886)
Supplement: Supplementary Information [file srep15886-s1.pdf]

SUPPLEMENTARY INFORMATION FOR

The evolution of tumor metastasis during clonal expansion with alterations in metastasis driver genes

Kimiyo N. Yamamoto[1], Akira Nakamura[2], and Hiroshi Haeno[1*]

[1]Department of Biology, Faculty of Sciences, Kyushu University, Fukuoka 812-8581, Japan.

[2]Department of Radiation Oncology and Image-Applied Therapy, Kyoto University, Kyoto, Japan.

[*]Author for correspondence. Email: haeno@kyushu-u.org (H.H.)

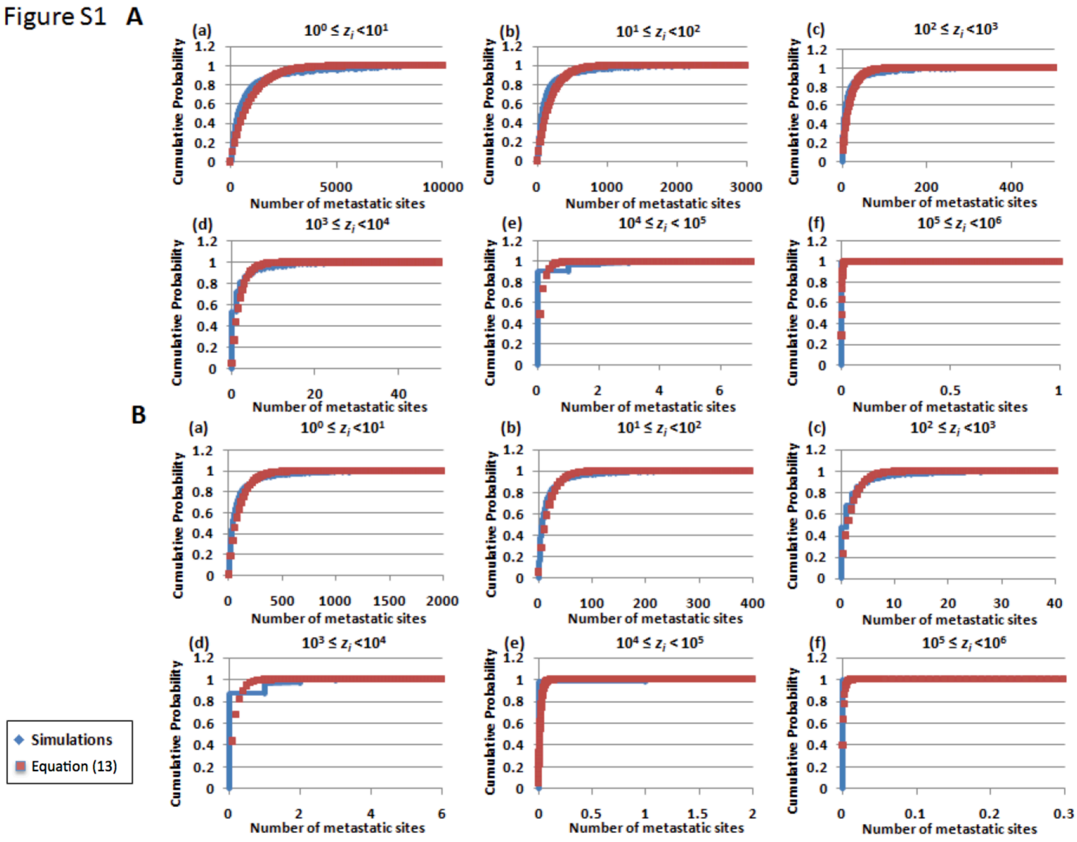

Figure S1. Exponential distribution of the number of metastatic sites generated from type-2 cells in different size categories.

Blue lines denote the results of direct computational simulation and red lines denote a cumulative exponential distribution with the mean of the expected numbers of the categories. Parameter values are (A) r=1.0, a_1=1.5, a_2=2.0, a_3=2.0, u_1=u_2=0.001, q=0.01, and M_1=10^7; and (B) r=1.0, a_1=1.5, a_2=2.0, a_3=2.0, u_1=u_2=0.001 q=0.001, and M=10^7.

Figure S2

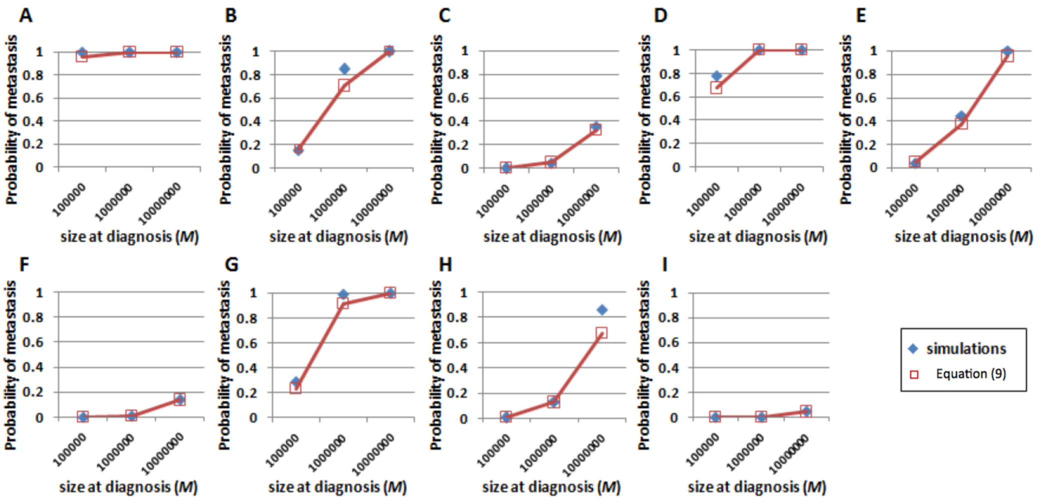

J

Panel \ Parameter	r	a_1	a_2	a_3	$u_1=u_2$	q
A	1	1.5	2	2	0.01	0.01
B					0.001	
C					0.0001	
D					0.01	0.001
E					0.001	
F					0.0001	
G					0.01	0.0001
H					0.001	
I					0.0001	

*death rate of each cell type is 100 times smaller than its growth rate

Figure S2. Probability of metastasis generated from type-2 cells with various (epi)genetic alterations and metastatic rates.

The figures show the dependence of the probability of the existence of metastatic cells (type-3 cells) upon diagnosis for various parameters. The blue dots show the results of the direct computer simulations, while the red line shows the predictions of the analytical approximations (Eq. (9)). Parameter values are listed in panel J.

Figure S3

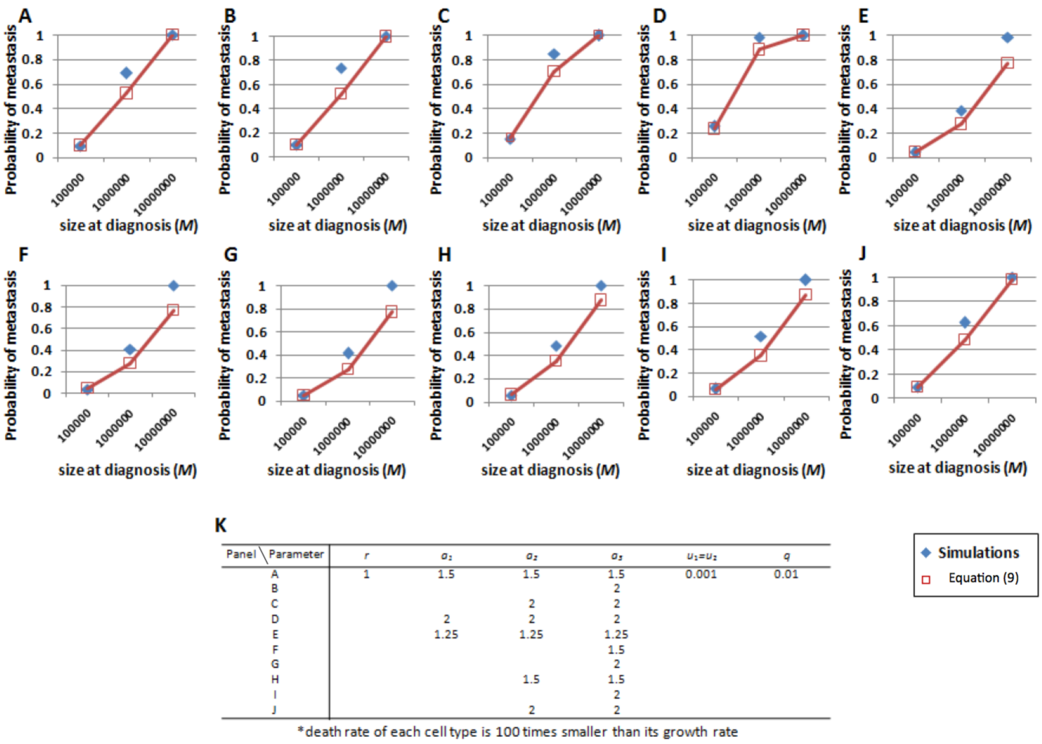

K

Panel \ Parameter	r	a_1	a_2	a_3	$u_1=u_2$	q
A	1	1.5	1.5	1.5	0.001	0.01
B				2		
C			2	2		
D		2	2	2		
E		1.25	1.25	1.25		
F				1.5		
G				2		
H			1.5	1.5		
I				2		
J			2	2		

*death rate of each cell type is 100 times smaller than its growth rate

Figure S3. Probability of metastasis generated from type-2 cells with various growth rates for each cell type.

The figures show the dependence of the probability of the existence of metastatic cells (type-3 cells) upon diagnosis on the growth rate for each cell type. The blue dots show the results of the direct computer simulations, while the red line shows the predictions of the analytical approximations (Eq. (9)). Parameter values are listed in panel K.

Figure S4

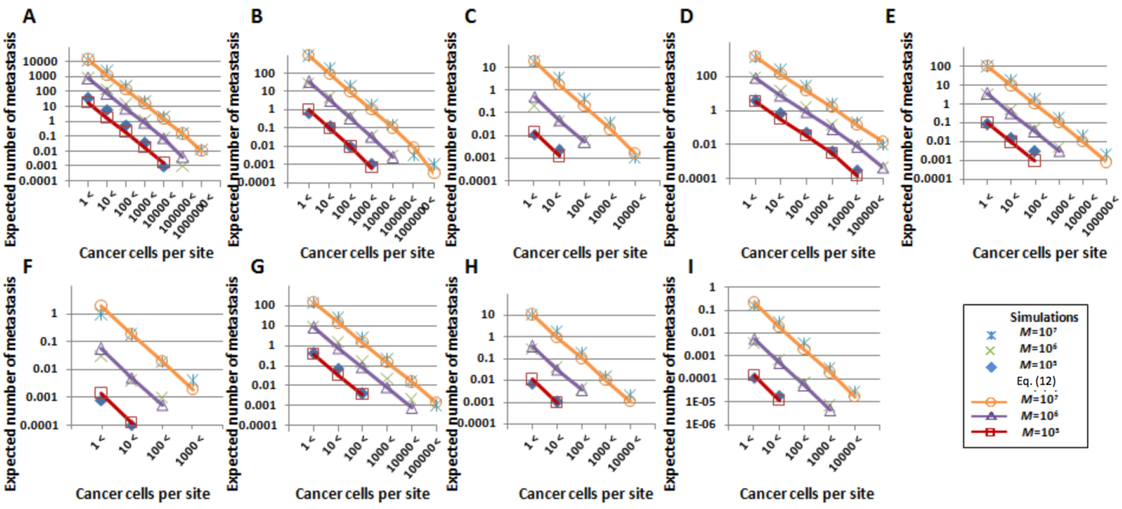

J

Panel\Parameter	r	a_1	a_2	a_3	$u_1=u_2$	q
A	1	1.5	2	2	0.01	0.01
B					0.001	
C					0.0001	
D					0.01	0.001
E					0.001	
F					0.0001	
G					0.01	0.0001
H					0.001	
I					0.0001	

*death rate of each cell type is 100 times smaller than its growth rate

Figure S4. Expected number of metastases generated from type-2 cells with various (epi)genetic alterations and metastatic rates.

The figures show the dependence of the expected number of metastatic sites at diagnosis on various parameters. Dots show the results of the direct computer simulations, while lines show the predictions of the analytical approximations (Eq. (12)). Parameter values used are listed in panel J.

Figure S5

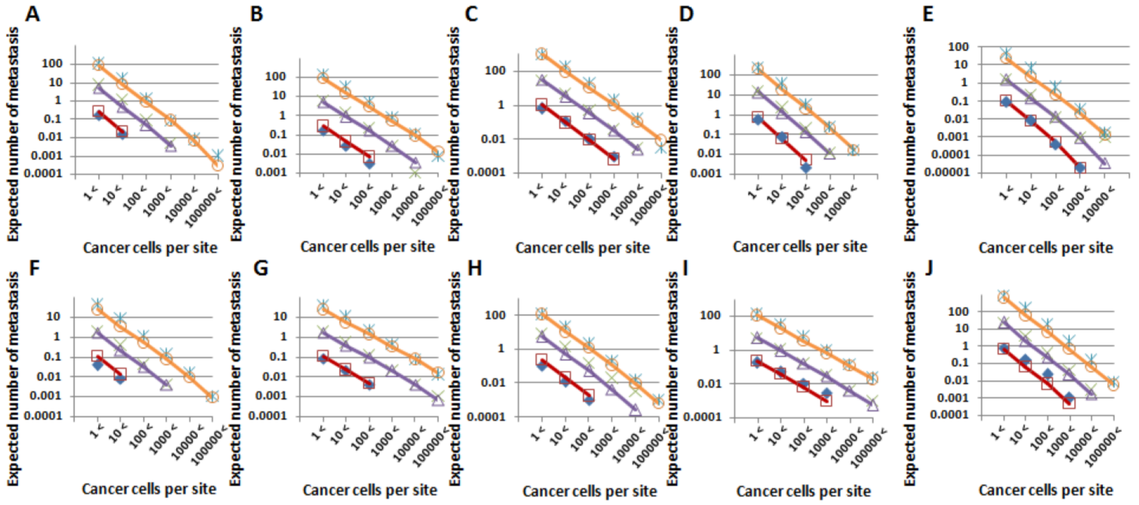

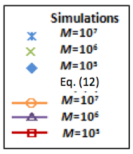

K

Panel \ Parameter	r	a_1	a_2	a_3	$u_1=u_2$	q
A	1	1.5	1.5	1.5	0.001	0.01
B				2		
C			2	2		
D		2	2	2		
E		1.25	1.25	1.25		
F				1.5		
G				2		
H			1.5	1.5		
I				2		
J			2	2		

*death rate of each cell type is 100 times smaller than its growth rate

Figure S5. Expected number of metastases generated from type-2 cells with various growth rates for each cell type.

The figures show the dependence of the expected number of metastatic sites upon diagnosis on growth rate for each cell type. Dots show the results of the direct computer simulations, while lines show the predictions of the analytical approximations (Eq. (12)). Parameter values used were listed in panel K.

Figure S6

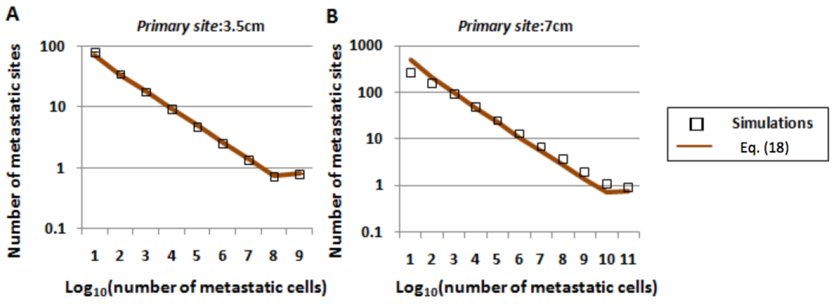

Figure S6. Expected number of metastases generated from type-1 and type-2 cells. The figures show the number of metastatic sites of the indicated sizes upon diagnosis on the *x*-axis based on computational simulations (black squares) and theoretical formulas (brown lines) when the primary tumor is diagnosed at a diameter of (A) 3.5 cm and (B) 7 cm. Parameter values are r=0.11, d=0.01r, a_1=0.16, b_1=0.01a_1, a_2=0.24, b_2=0.01a_2, a_3=0.58, b_3=0.01a_3, u_1=u_2=6.31×10^{-5}, and q=q'=6.31×10^{-7}, which are based on the estimation using time series clinical data in the pancreatic cancer autopsy program [1].

Figure S7

A ***Primary site*: 1cm**

	type-1	type-2
1-10	1.175936764	0.034177394
10^1-10^2	0.573806014	0.01209167
10^2-10^3	0.318961756	0.004930983
10^3-10^4	0.169839703	0.001912739
10^4-10^5	0.090031486	0.000738073
10^5-10^6	0.047703939	0.00028463
10^6-10^7	0.025275195	0.000109738
10^7-	0.02848195	6.86675E-05

B ***Primary site*: 2cm**

	type-1	type-2
1-10	13.81095788	0.913194
10^1-10^2	6.739146982	0.323081
10^2-10^3	3.746092067	0.131753
10^3-10^4	1.994706742	0.051108
10^4-10^5	1.057387687	0.019722
10^5-10^6	0.560265746	0.007606
10^6-10^7	0.296848151	0.002934
10^7-10^8	0.334517372	0.001841

C ***Primary site*: 3cm**

	type-1	type-2
1-10	44.10934641	6.053571
10^1-10^2	21.52344329	2.141703
10^2-10^3	11.9642442	0.873389
10^3-10^4	6.370681267	0.338793
10^4-10^5	3.377077836	0.130736
10^5-10^6	1.789373054	0.050423
10^6-10^7	0.948071671	0.019446
10^7-10^8	0.502318869	0.0075
10^8-10^9	0.266144576	0.002892
10^9-	0.407156463	0.004008

D ***Primary site*: 4cm**

	type-1	type-2
1-10	86.76831124	22.77998
10^1-10^2	42.33916343	8.059366
10^2-10^3	23.53508607	3.286618
10^3-10^4	12.53188496	1.2749
10^4-10^5	6.643112279	0.491967
10^5-10^6	3.519908833	0.189744
10^6-10^7	1.864969322	0.073178
10^7-10^8	0.988120739	0.028222
10^8-10^9	0.523537918	0.010884
10^9-	0.577246722	0.005756

E ***Primary site*: 5cm**

	type-1	type-2
1-10	132.6275931	62.98688
10^1-10^2	64.71649914	22.28423
10^2-10^3	35.97398375	9.087534
10^3-10^4	19.15530815	3.525112
10^4-10^5	10.1541678	1.360295
10^5-10^6	5.380271089	0.524645
10^6-10^7	2.850653526	0.202337
10^7-10^8	1.510367938	0.078034
10^8-10^9	0.800241159	0.030095
10^9-	0.90179028	0.018892

F ***Primary site*: 6cm**

	type-1	type-2
1-10	174.3546853	143.476
10^1-10^2	85.07750594	50.76061
10^2-10^3	47.29206395	20.70023
10^3-10^4	25.18192215	8.029752
10^4-10^5	13.34885668	3.098576
10^5-10^6	7.073003818	1.195073
10^6-10^7	3.747521814	0.460897
10^7-10^8	1.985557608	0.177751
10^8-10^9	1.052011819	0.068552
10^9-	1.185510398	0.043034

Figure S7. The number of metastatic sites generated from type-1 and type-2 cells in different size categories.

The table shows the number of metastatic sites generated by type-1 and type-2 cells in various size categories. The diameter at diagnosis is (A) 1 cm, (B) 2 cm, (C) 3 cm, (D) 4 cm, (E) 5 cm, and (F) 6 cm. Parameter values are based on the estimation in the autopsy program [1]; r=0.11, d=0.01r, a_1=0.16, b_1=0.01a_1, a_2=0.24, b_2=0.01a_2, a_3=0.58, b_3=0.01a_3, u_1=u_2=6.31×10^{-5}, and q=q'=6.31×10^{-7}.

Figure S8

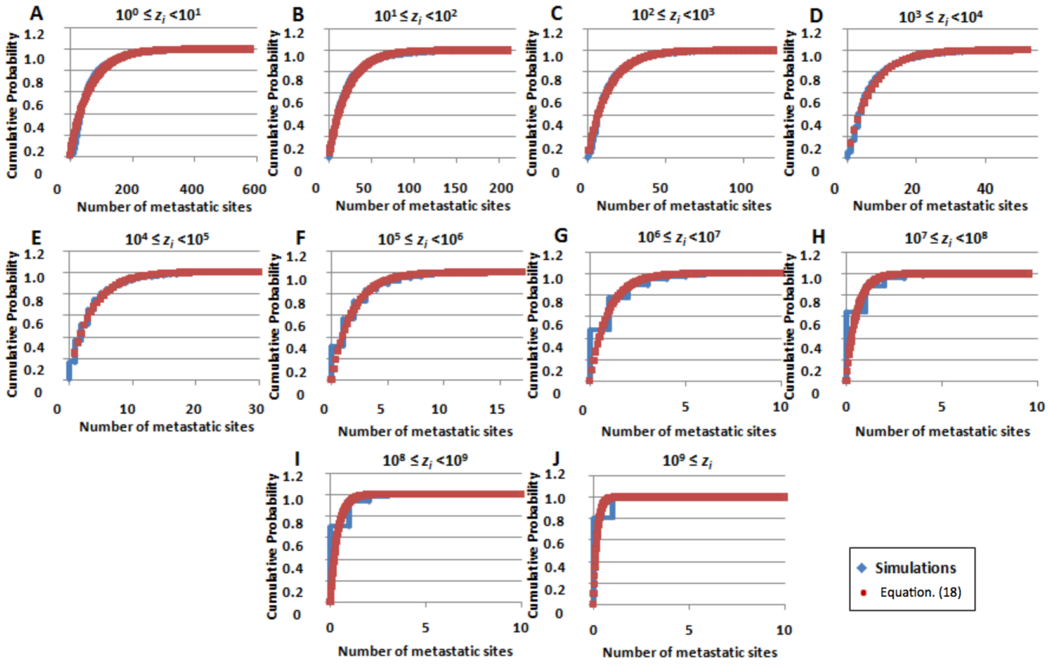

Figure S8. Exponential distribution of the number of metastatic sites generated from both type-1 and type-2 cells in different size categories.

Blue lines denote the results of direct computational simulation, and red lines denote an exponential distribution with the mean of the expected numbers of the categories when the size of the primary tumor is 1 cm. Parameters are based on the estimation in the autopsy program [1].; r=0.11, d=0.01r, a_1=0.16, b_1=0.01a_1, a_2=0.24, b_2=0.01a_2, a_3=0.58, b_3=0.01a_3, u_1=u_2=6.31x10^{-5}, q=q'=6.31×10^{-7}, and M=2.7×10^9.

Figure S9

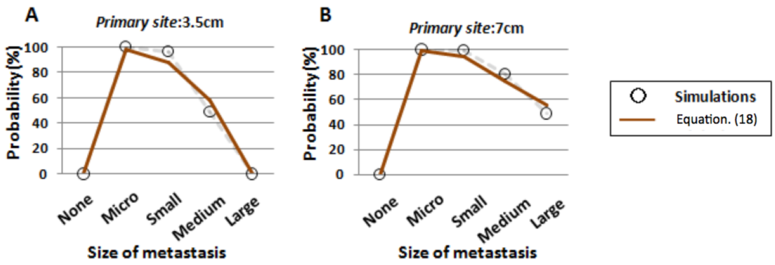

Figure S9. Probability of metastasis generated from both type-1 and type-2 cells in different size categories.

Panels show the probabilities of existence of metastases of the indicated sizes upon diagnosis on the *x*-axis, with the assumption that the number of metastatic sites follows an exponential distribution with the mean of the expected numbers of categories. “Large” denotes a metastatic site larger than 2.5 cm, “medium” denotes between 0.5 cm and 2.5 cm, “small” denotes between 0.1 cm and 0.5 cm, and “micro” denotes less than 0.1 cm. The diameter of the primary tumor at diagnosis is (A) 3.5 cm and (B) 7 cm. Parameter values are based on the estimation in the autopsy program [1].; r=0.11, d=0.01r, a_1=0.16, b_1=0.01a_1, a_2=0.24, b_2=0.01a_2, a_3=0.58, b_3=0.01a_3, u_1=u_2=6.31×10^{-5}, and q=q'=6.31×10^{-7}.

Figure S10

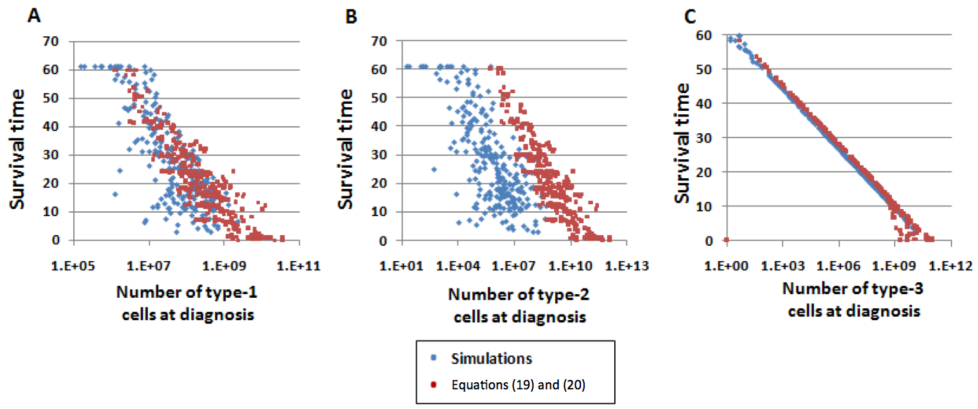

Figure S10. Relationship between the number of cells in each population at diagnosis and survival duration.

Red dots show the theoretical predictions by Eqs. (19) and (20), and blue dots show the results of computational simulations. Population types are (A) type-1, (B) type-2, and (C) type-3. Parameter values are based on the estimation in the autopsy program [1].; r=0.11, d=0.01r, a_1=0.16, b_1=0.01a_1, a_2=0.24, b_2=0.01a_2, a_3=0.58, b_3=0.01a_3, u_1=u_2=6.31×10^{-5}, q=q'=6.31×10^{-7}, M=10$^{N(9.47,0.59)}$, and M'=10$^{11.2}$. Here, N(9.47, 0.59) represents the normal distribution with mean 9.47 and variance 0.59.

Figure S11

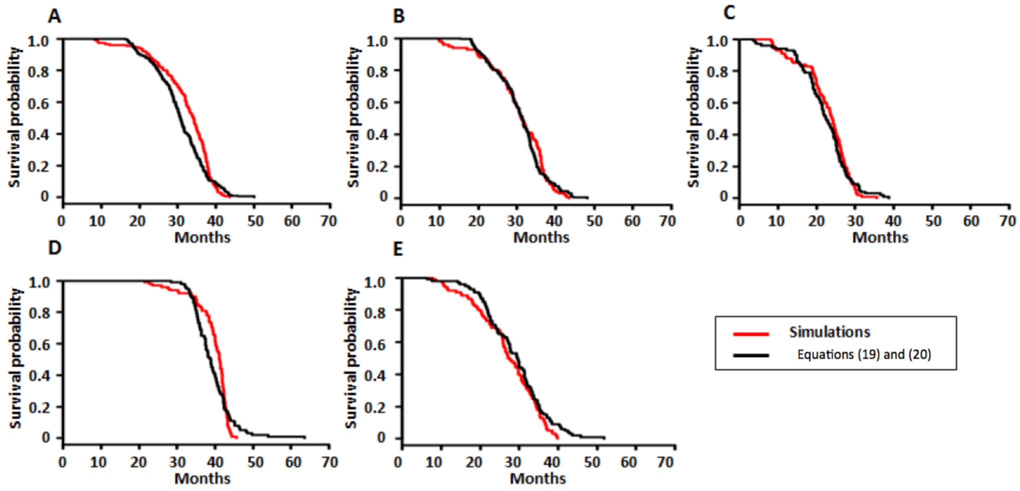

F

Panel \ Parameter	r	a_1	a_2	a_3	u_1=u_2	q	M	M'
A	1	1.5	2	2	0.01	0.001	1000000	100000000
B	1	1.5	1.5	2	0.01	0.001	1000000	100000000
C	1	1.5	1.5	2	0.01	0.001	10000000	1000000000
D	1	2	2	2	0.01	0.001	1000000	100000000
E	1	1.25	1.5	2	0.01	0.001	1000000	100000000

*death rate of each cell type is 100 times smaller than its growth rate

Figure S11. Survival based on computational simulations and theoretical predictions.

Survival analyses comparing theoretical predictions (black lines, Eq. (19) and (20)) and computational simulations (red lines). P values are (A) 0.10, (B) 0.92, and (c) 0.86, (D) 0.77, and (E) 0.10. Parameter values used are listed in panel F.

REFERENCES

1. Haeno H, Gonen M, Davis MB, Herman JM, Iacobuzio-Donahue CA, Michor F. Computational modeling of pancreatic cancer reveals kinetics of metastasis suggesting optimum treatment strategies. Cell 2012:148: 362-375.
